# Additional Society Meetings

**Published:** 1915-10

**Authors:** 


### Additional Society Meetings.

---

**The American Roentgen Ray Society,** Atlantic City, Sept 22 to 25. The meeting opened with papers on the subject of teaching Roentgenology to the undergraduate. The lack of system in placing the subject in the curriculum was shown. In some schools it came under surgery, in others under therapeutics, medicine and practically under every course on the list. The papers were read by Drs. Zapffe, of Chicago, Cole. of New York, and Pancoast, of Philadelphia.

Oral sepsis as a Focus of Infection by Dr. Darling of New York, brought out the well-known relation of oral sepsis to inflammatory conditions in remote parts of the body, joints, etc. It was demonstrated by Dr. Childs of Denver that in a great many fractures about the joints, better functional end results are obtained with surgical interference. Some lantern slides of roentgenography showing a considerable amount of fragment displacement proved his contention.

Prof. Shearer, of Ithaca, read a paper on "The Physical

Aspect of Roentgen Ray Measurement and Dosage" which was of great interest to roentgenologists.

Dr. Coolidge, the inventor of the Coolidge tube, gave in detail the results of his investigations and experiments. He saw tremendous discoveries clear ahead within striking distance in the matter of perfecting sources of Roentgen rays. He made a plea for the standardization of apparatus used by roentgenologists.

Dr. Dunham, of Cincinnati, showed a very interesting collection of slides of lung diseases. He emphasized the fact that an X-Ray plate properly taken and interpreted is the means of preference in diagnosticating incipient tuberculosis.

The Roentgenographic Diagnosis of Metastatic Malignancy in the Lung, was the subject of a paper by Drs. Moore and Carman, of Rochester, Minn. Lantern slides from X-Ray plates taken of the lungs of patients suffering from carcinoma and sarcoma in various parts of the body demonstrate the metastatic processes in the lungs. It was brought out that all cases of carcinoma of the heart should have a roentgenograph of the chest taken before operation and as soon as possible following operation thorough roentgen ray therapy should be applied. In this way 83 per cent. of symptomatic cures have been obtained.

Dr. George, of Boston, showed that with proper technique, of exposure, position, etc., and interpretation of plates a greater percentage of gall stones are diagnosed by the roentgen method.

Dr. Gray, president of the society, gave a brief review of the progress of Roentgenology.

Dr. Wm. L. Rodman, president of the A. M. A., said that he for one, appreciates the assistance of the Roentgenologists in the diagnosis of early gastric cancer. The X-Rays are to be preferred to other methods for in a large number of those cases there are no clinical means of making a diagnosis—achylic faeces, acid, etc., are late manifestations.

Comparison as to the value of radium and roentgen rays was treated in papers read by Drs. Newcomet, of Philadelphia; Holding, of New York, and Boggs, of Pittsburgh. Radium was of value when the amount of tissues to be penetrated was under 2.5 c.m. It was apt to stimulate growth at periphery of tumor. Tumors exceeding 2.5 c.m. should receive supplementary roentgen ray therapy, 300 to 500 X units are required to destroy cancer cells. It is often necessary to give 3,000 X through numerous skin areas, cross-firing to give a deep growth the desired destruction dose. At times results are obtained with X-Rays where radium fails and vice versa.

In exophthalmic goitre 50 per cent. cures thymic and thyroid gland are rayed—new technique.

In the treatment of menorrhagia, Dr. Lange, of Cincinnati, reported 50 cases, 100 per cent. cures; age of patients, 17 to 56; often one or two treatments sufficient.

Wonderful results are obtained with X-Ray therapy in Myomata.

Of special interest were the following papers:

Studies in the Normal and Pathological Anatomy and Physiology of the Digestive Tract of Children, by Dr. L. T. LeWald of New York.

Text books teach stomach of infants tubular and vertical to body. Roentgenography of numerous infants, 3 days to seven years of age, show stomach as in adult, often extending laterally from ribs on one side to ribs on other, especially when gas inflated, the principal cause of which is from swallowing air.

New Roentgen Evidence of the Early Diagnosis of Early Gastric Carcinoma, by Dr. A. W. George, of Boston.

This evidence consisted of a contraction ring about the pyloris when malignant change starts from a pre-existing ulcer.

Syphilis of the Stomach, by Dr. L. T. LeWald, of New York.

Stomach shadow simulates shadow of gastric cancer but lacks ''finger print'' mark. Wasserman reaction will assist in differentiating.

Roentgenologic Diagnosis of Duodenal Ulcer, by Dr. R. D. Carman, of Rochester, Minn. A very strong presentation of the subject.

The next paper, by Dr. J. T. Case, of Battle Creek, Mich., supplements the above. His slides interesting in showing diverticula of duodenum.

A manufacturers' exhibit showing all the latest models of apparatus and appliances was very interesting and instructive to the roentgenologists.

Reported by Dr. J. M. Garratt, Buffalo.

